# The genetics of cross-sectional and longitudinal body mass index

**DOI:** 10.1186/1471-2156-4-S1-S14

**Published:** 2003-12-31

**Authors:** Lisa Strug, Lei Sun, Mary Corey

**Affiliations:** 1 University of Toronto, Public Health Sciences, 12 Queen's Park Crescent West, Toronto, Ontario, Canada; 2 The Hospital for Sick Children, 555 University Avenue, Toronto, Ontario, Canada

## Abstract

There has been a lack of consistency in detecting chromosomal loci that are linked to obesity-related traits. This may be due, in part, to the phenotype definition. Many studies use a one-time, single measurement as a phenotype while one's weight often fluctuates considerably throughout adulthood. Longitudinal data from the Framingham Heart Study were used to derive alternative phenotypes that may lead to more consistent findings. Body mass index (BMI), a measurement for obesity, is known to increase with age and then plateau or decline slightly; the decline phase may represent a threshold or survivor effect. We propose to use the weight gain phase of BMI to derive phenotypes useful for linkage analysis of obesity. Two phenotypes considered in the present study are the average of and the slope of the BMI measurements in the gain phase (gain mean and gain slope). For comparison, we also considered the average of all BMI measurements available (overall mean). Linkage analysis using the gain mean phenotype exhibited two markers with LOD scores greater than 3, with the largest score of 3.52 on chromosome 4 at ATA2A03. In contrast, no LOD scores greater than 3 were observed when overall mean was used. The gain slope produced weak evidence for linkage on chromosome 4 with a multipoint LOD score of 1.77 at GATA8A05. Our analysis shows how omitting the decline phase of BMI in the definition of obesity phenotypes can result in evidence for linkage which might have been otherwise overlooked.

## Background

Body mass index (BMI = weight (kg)/height^2 ^(m)) is a commonly used estimate of adiposity that correlates well with more direct and invasive measures of percentage body fat. Since height and weight are collected in many studies, the convenience and cost-effectiveness of using BMI leads to its use as a quantitative trait in linkage studies of obesity. Unfortunately, finding the chromosomal locations of genes responsible for controlling BMI has proven difficult [1]. The current study hypothesizes that the lack of consistency may reflect the large variability in the phenotype definition, since many studies use a single measurement of BMI that may fluctuate considerably throughout adulthood.

The phenotypic definition of obesity is often a result of the study design, leaving little choice for more robust alternatives. Study designs for quantitative trait locus (QTL) mapping of BMI-related phenotypes vary substantially, from longitudinal [2-4] to cross-sectional [5] designs. BMI-based phenotypes include the maximum observed value [3], the last value observed [4], the only value observed [5], and the value at a specific age [2]. The populations considered in different studies may also differ, concentrating for example on a young population [3], an older one [4], one that spans all ages [2], and even one that is known to exhibit a high prevalence of type 2 diabetes mellitus [3]. The longitudinal data from the Framingham Heart Study can help determine more robust phenotypic definitions of BMI for use in genetic studies whether they be longitudinal or cross-sectional in design.

In a technical report by (Bellhouse DR, Chipman HA, and Stafford JE, 2002) from the Ontario Health Study (OHS), analysis of cross-sectional survey data provided significant evidence of a nonlinear association between BMI and age, characterized by an increasing phase to approximately age 55 followed by a slight decline as subjects aged. The decline phase may reflect both a survivor effect as well as a true threshold effect. The existence of such a nonlinear relationship between BMI and age implies that, for studies of obesity-related traits, any phenotype that incorporate BMI measurements across the whole age range (gain and decline phases), whether they be cross-sectional phenotypes (a mean or single measurement) or longitudinal (a slope), would be biased by the decline phase. As an alternative, we derive two obesity phenotypes based on BMI measurements in the gain phase only (*gain mean *and *gain slope*) and evaluate them in a genome-wide linkage analysis.

## Methods

**Figure 1:**
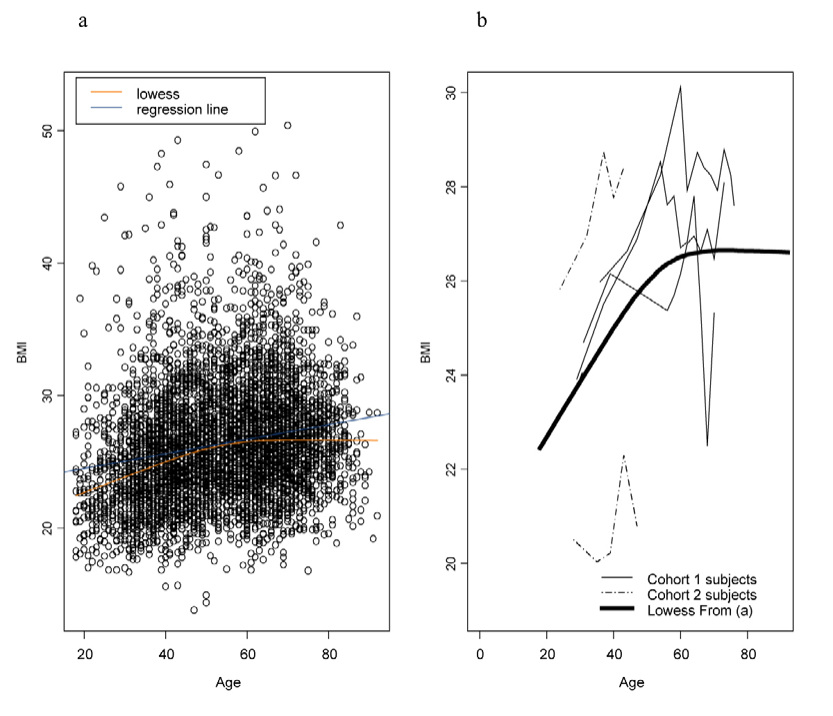
**The BMI relationship with age **Cross-sectional and longitudinal BMI by age relationship (a) raw data sample of 5000 observations with lowess smoother and regression line (b) individual profiles in one family superimposed on the lowess smoother from (a).

The Framingham Heart Study data, supplied to the participants of Genetic Analysis Workshop 13, was used to explore longitudinal BMI as a phenotype for genetic analysis. Observations taken when age < 18 years were excluded. The data indicated a nonlinear relationship between age and BMI, one quite similar to that described in the OHS. Plots of individuals' BMI by age exhibited the existence of a consistent nonlinear profile within individuals. Figure 1 illustrates the cross-sectional and longitudinal aspect of the general relationship between BMI and age observed in the data, as well as the variability of individual measurements.

Within individuals, on average, BMI increased from age 18 until age 53 and then began to decline or stabilize. Depending on the age range of the individual during data collection, different components of this profile might be seen. For this reason and to address issues of nonlinearity, each individual's BMI by age profile was summarized by two phases: a *gain phase *and a *decline phase*, demarcated by the observed *age at maximum BMI*. One could then define BMI phenotypes derived solely from an individual's *gain phase*, which would then have a simple linear relationship with age. Two such possibilities would be 1) a cross-sectional phenotype defined as the average of the BMI measurements in the gain phase, *gain mean*, measuring the tendency to be heavier-set, and 2) a longitudinal phenotype, the slope in the gain phase, *gain slope*, measuring the rate of weight gain.

The *gain slope *for an individual was calculated as the coefficient for age from a simple linear model, regressing BMI on age, with the data restricted to that observed in the individual's gain phase. Thus, both the *gain mean *and the *gain slope *utilized the longitudinal nature of the data to obtain more reliable measures of cross-sectional and longitudinal BMI phenotypes for obesity-related traits.

For individuals to have a measurement for *gain mean *and *gain slope*, it was required that they have at least three BMI measurements in their gain phase. This choice was arbitrary and made by the authors in an attempt to reduce the noise in the measurements while still ensuring sufficient subjects were included in the analysis. If a height measurement was missing at a particular visit for an individual whose data would otherwise be complete at that visit, height was imputed from other visits. Specifically, the measurement from the closest gain phase visit, with height information available, was used.

A natural log transformation was used to achieve approximate normality for the phenotypic distributions. The heritability and significance of covariate information in the additive genetic model for the *gain mean *and *gain slope *were evaluated and then QTL mapping using SOLAR 1.6.7 [6] was used to perform additive variance component two-point linkage analyses. Information content for the markers was calculated using GENEHUNTER (version 2.1_r2 beta) [7]. Heritability calculations, covariate screening, and linkage analysis were also performed on the *overall mean*, a BMI phenotype defined as the average of all the BMI measurements including the decline phase of the individuals. Approximate multipoint linkage analyses [6] of the *gain slope *and *gain mean *phenotypes were also conducted. All genetic models used for linkage analysis included gender, cohort (whether they came from the original Cohort 1 or the offspring Cohort 2 of the Framingham Heart Study data, where Cohort 2 was generally younger with fewer visits) and the cohort-by-gender interaction. The two-point LOD scores from the linkage analyses of *gain mean *and *overall mean *were compared to evaluate the utility of omitting the decline phase when defining a BMI phenotype.

## Results

Table 1 provides descriptive statistics for the *age at maximum BMI*, the *gain slope*, the *gain mean*, and the *overall mean*. The majority of subjects in the study were defined to have both gain phase phenotypes (*N *= 2226 of 2878 for whom BMI data was available in 330 pedigrees); 375 individuals had only a decline phase, with their maximum BMI measurement at their first observation; 277 individuals had only one BMI measurement before their *age at maximum BMI*. The *gain slope *appeared quite variable across individuals. The distributions of *gain mean *and *overall mean *were quite similar. The variability of the *age at maximum BMI *was large (i.e., large standard deviation).

All the phenotypic models, accounting for the covariates, displayed significant heritability, with the heritability (standard error) for the *gain slope, gain mean*, and *overall mean *equal to 0.11 (0.04), 0.49 (0.04), and 0.49 (0.03), respectively.

All markers exhibiting two-point LOD scores greater than 2 for *gain mean ***or ***overall mean *are listed in Table 2 with their flanking marker information in parentheses. The information content for the markers is also provided. For each of the markers listed in the table, the LOD score for *gain mean *was larger than that for *overall mean*. There were two markers for *gain mean *with LOD scores greater than 3, the largest being 3.52 located on chromosome 4 at 93 cM. The largest two-point LOD score observed for *overall mean *was 2.49 on chromosome 9 at 92 cM. The LOD score at this marker for *gain mean *was 3.14.

**Figure 2:**
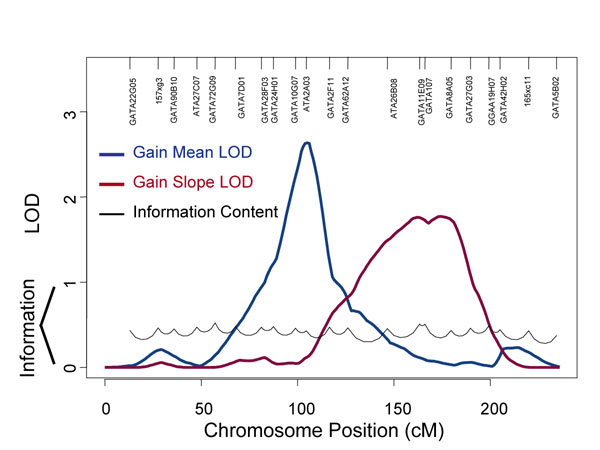
**Multipoint analysis of both *gain phase phenotypes *on chromosome 4.** Multipoint results for the *gain slope *(in red) and the *gain mean *(in blue) along with the information content for this chromosome (in black).

The two-point evidence of linkage for the *gain slope *was weak, with only four markers across the genome exhibiting LOD scores greater than 1, the largest being 1.29 on chromosome 4 at 158 cM. However, three of these markers were in the same general region on chromosome 4 (130 cM, 143 cM, and 158 cM). Table 3 lists the two-point LOD scores, map locations and information content for these three markers, with the flanking marker results in parentheses. Due to the additional flanking marker evidence of weak linkage for the *gain slope *and the lack of evidence at locations in any other region or on any other chromosome, approximate multipoint analysis was also performed. The multipoint analysis produced larger LOD scores on chromosome 4 with the largest LOD score being 1.77 at 174 cM. The multipoint results for the *gain slope *on chromosome 4 are illustrated in Figure 2.

**Figure 3:**
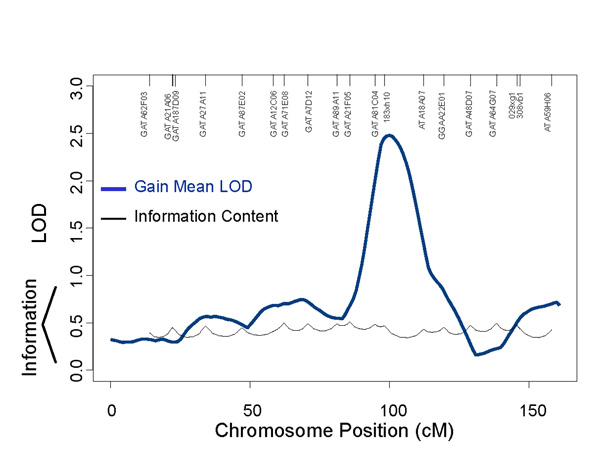
**Multipoint analysis of the Gain Mean on chromosome 9. **Multipoint results for the gain mean (in blue) along with the information content for this chromosome (in black).

Figure 2 also provides the multipoint results for the *gain mean *phenotype on chromosome 4 and the information content across this chromosome. The largest multipoint result for the *gain mean *was also on chromosome 4 with a LOD score of 2.64 at 105 cM. The multipoint results for the *gain mean *phenotype on chromosome 9, the other chromosome that provided a two-point LOD score for the *gain mean *that was greater than 3, are illustrated in Figure 3 along with the information content across this chromosome. The largest multipoint LOD score on this chromosome was 2.48 at 100 cM. The multipoint LOD scores for the *gain mean *phenotype were smaller than those observed in the two-point analysis. The information content across chromosomes 4 and 9 was low (less than 0.52 for all markers on each of these chromosomes).

## Discussion

The *gain slope*, *gain mean *and *overall mean*, were shown to be significantly heritable, with the phenotypes based on mean values exhibiting much larger heritability estimates than the *gain slope*. The most apparent gain from considering the nonlinear relationship in the definition of the BMI phenotype can be seen in the comparison of LOD scores for *gain mean *and *overall mean*. Namely, it appears that linkage analysis based on the *gain mean *phenotype provided us with possible chromosomal locations influencing an individual's tendency to be heavier-set, while the analysis using the *overall mean *phenotype (including both gain and decline phases) did not produce strong linkage evidence at these potential locations. The *gain mean *phenotype provided two regions with two-point LOD scores greater than 3 with no such regions for the *overall mean *phenotype. The proximity of the two elevated LODs for the adjacent markers on chromosome 9 provided additional evidence that this location is worthy of future study. Moreover, the use of the *gain mean *phenotype detected chromosomal locations that have already been implicated in previous studies using more direct measures of the obesity phenotype.

The Québec Family Study [8], a genome-wide scan, found nine QTLs affecting abdominal subcutaneous fat, two of which were on chromosomes 4 and 9. The region on chromosome 9 reported in Table 2 is the same region (*D*9*S*257 at 92 cM) reported in the Québec Family Study to influence abdominal subcutaneous fat.

A study of Pima Indians [3], who have a high prevalence of both type 2 diabetes and obesity, performed genome scans for loci linked to type 2 diabetes and obesity. Variance components linkage analyses were conducted on sibships. Phenotypic information came from the participants of their original longitudinal study, measuring the age at onset of type 2 diabetes. The mean age at onset of diabetes among affected offspring was 34 years (SD = 10.6) and the mean age at last examination of nondiabetic offspring was 35.5 years (SD = 11.1). The maximum BMI observed in the study period was used as an individual's BMI phenotype, and the largest associated LOD score was on chromosome 11 in the same region as that found in the current study (*157xh6 *at 131 cM).

Only weak linkage signals were observed in our study for the *gain slope*. No chromosomal locations linked to a slope phenotype were reported in the Human Obesity Map [1]. However, it is interesting to note that the signals observed in the current study were in the same general region on chromosome 4 and did increase in size in the multipoint analysis. This region is approximately 20 cM, calculated using the Marshfield Map [9], from the location reported for chromosome 4 in the Québec Family Study [7] (*D*4*S*2417), which has been suggested to contain a potential candidate gene.

The only linkage results for obesity reviewed in the Human Obesity Map [1] that corresponded to the regions found in the current analysis were those from the Québec Family Study [8] and the site on chromosome 11 found by Hanson et al. [3], who studied a relatively young sample that would, presumably, have not yet entered their decline phase. A measure such as abdominal subcutaneous fat used in the Québec Family Study is a more accurate measure of obesity. However, such a direct and invasive measure is only available in studies by design. Accounting for the nonlinearity of BMI by using the *gain mean *of BMI, when longitudinal data is available, seems to be a practical and simple alternative. The *gain mean *is not plagued by the fluctuations of a single cross-sectional measurement yet it is still easily calculated from the height and weight data that is often collected in studies with an alternative focus. The *gain mean *may also increase the power to detect linkage to obesity by removing the decline phase of individuals, which appears to be introducing competing sources of variation.

In cross-sectional study designs one might incorporate the results of this study by choosing to restrict BMI phenotypes on the basis of age; selecting the measurement for the BMI phenotype to be at ages less than the lower confidence limit for the average *age at maximum BMI*. The current study observed a large standard deviation for the *age at maximum BMI. *This may not reflect the true population standard deviation, because an individual's *age at maximum BMI *had to occur at a particular point in time (i.e., study visit). Additionally, the data available on some individuals did not consist of data in both the gain and decline phases. For example, if data were available for an individual only in their gain phase then their *age at maximum BMI *may have been underestimated. However, when choosing a BMI phenotype in a cross-sectional study, with the intent to omit decline phase measurements, the possibility of a large standard deviation in the *age at maximum BMI *should be considered.

It is unknown how prevalent the use of a slope phenotype is for studying obesity-related traits, because there has been little discussion of these phenotypes in the published literature and no slope phenotypes exhibiting linkage were reported in the Human Obesity Map [1]. The *gain slope *may provide a means to distinguish genetic components controlling the rate of weight gain for an individual by omitting the decline phase in the definition of this phenotype. The analysis using the *gain slope *phenotype did suggest a potential region for future study, although the evidence, as measured by the LOD score, was weak. Inclusion of the decline phase in the definition of this phenotype might have led some, who have attempted to use slope phenotypes in past studies, to overlook this potential region.

## Conclusions

BMI is clearly an accessible surrogate measure for obesity and is available as a by-product of many studies with an alternative focus. Exploiting the nonlinearity between BMI and age to omit the decline phase of individuals in the definition of the phenotype proved fruitful in the current study; the *gain mean *phenotype exhibited higher LOD scores than the *overall mean *phenotype, some at loci that have already been implicated in a study using a more direct measure of obesity [8]. Furthermore, the *gain slope *phenotype exhibited some weak evidence for linkage while no such evidence for other obesity slope phenotypes have been previously reported [1]. The design implications of this study are, however, the most important finding. The methodology introduced here may be generalizable to many traits for which a similar temporal relationship is known to exist.

## Figures and Tables

**Table 1:** Summary statistics for the derived phenotypes and *age at maximum BMI*

**Phenotype**	**1st Quartile**	**Median**	**Mean**	**3rd Quartile**	**Standard Deviation**	**Sample Size**
*age max BMI*	44.00	52.00	52.78	63.00	13.52	2878
*gain slope*	0.08	0.16	0.19	0.26	0.17	2226
*gain mean*	23.63	25.99	26.48	28.54	4.23	2226
*overall mean*	23.36	25.81	26.26	28.42	4.29	2878

**Table 2:** Two-point linkage results for the cross-sectional BMI phenotypes

**Chr #**	**Marker**	***Gain Mean *LOD Score**	***Overall Mean *LOD Score**	**Location (cM)**	**Information Content**
4	*ATA2A03*	3.52 (0.96,0.42)	1.98 (0.40,0.70)	93 (88,105)	0.43 (0.46,0.47)
8	*GATA23D06*	2.60 (0.27,1.53)	1.82 (0.00,0.22)	26 (22,37)	0.51 (0.51,0.46)
9	*GATA81C04*	2.28 (0.09,3.14)	1.78 (0.37,2.49)	89 (80,92)	0.48 (0.51,0.47)
9	*183xh10*	3.14 (2.28,0.28)	2.49 (1.78,0.08)	92 (89,104)	0.47 (0.48,0.43)
11	*157xh6*	2.36 (0.45,0.00)	0.49 (0.25,0.00)	131 (123,147)	0.46 (0.46,0.13)

Only those markers exhibiting LOD scores greater than 2 are listed in the table; their flanking marker information is in parentheses.

**Table 3:** Two-point linkage results on chromosome 4 for the *gain slope*

**Marker**	***Gain Slope *LOD Score**	**Location (cM)**	**Information Content**
*ATA26B08*	1.03 (0.30,1.09)	130 (114,143)	0.45 (0.46,0.51)
*GATA11E09*	1.09 (1.03,0.47)	143 (130,146)	0.51 (0.45,0.50)
*GATA8A05*	1.29 (0.47,0.32)	158 (146,168)	0.43 (0.50,0.45)

All markers on chromosome 4 exhibiting a LOD score greater than 1 are listed in the table with the flanking marker information in parentheses.
